# Hypothyroidism Causes Endoplasmic Reticulum Stress in Adult Rat Hippocampus: A Mechanism Associated with Hippocampal Damage

**DOI:** 10.1155/2018/2089404

**Published:** 2018-03-20

**Authors:** Alejandra Paola Torres-Manzo, Margarita Franco-Colín, Vanessa Blas-Valdivia, Marisol Pineda-Reynoso, Edgar Cano-Europa

**Affiliations:** ^1^Instituto Politécnico Nacional, Laboratorio de Metabolismo I, Departamento de Fisiología “Dr. Mauricio Russek Berman”, Escuela Nacional de Ciencias Biológicas, Av. Wilfrido Massieu s/n, Esq. Manuel L. Stampa, Unidad Profesional Adolfo López Mateos, Del. Gustavo A. Madero, 07738 Ciudad de México, Mexico; ^2^Instituto Politécnico Nacional, Lab. Neurobiología, Departamento de Fisiología “Dr. Mauricio Russek Berman”, Escuela Nacional de Ciencias Biológicas, Av. Wilfrido Massieu s/n, Esq. Manuel L. Stampa, Unidad Profesional Adolfo López Mateos, Del. Gustavo A. Madero, 07738 Ciudad de México, Mexico; ^3^Instituto Politécnico Nacional, Escuela Superior de Medicina, Plan de San Luis y Díaz Mirón s/n, 11340 Ciudad de México, Mexico

## Abstract

Thyroid hormones (TH) are essential for hippocampal neuronal viability in adulthood, and their deficiency causes hypothyroidism, which is related to oxidative stress events and neuronal damage. Also, it has been hypothesized that hypothyroidism causes a glucose deprivation in the neuron. This study is aimed at evaluating the temporal participation of the endoplasmic reticulum stress (ERE) in hippocampal neurons of adult hypothyroid rats and its association with the oxidative stress events. Adult Wistar male rats were divided into euthyroid and hypothyroid groups. Thyroidectomy with parathyroid gland reimplementation caused hypothyroidism at three weeks postsurgery. Oxidative stress, redox environment, and antioxidant enzyme markers, as well as the expression of the ERE through the pathways of PERK, ATF6, and IRE1, were evaluated at the 3rd and 4th weeks postsurgery. We found a rise in ROS and nitrite production; also, catalase increased and glutathione peroxidase diminished their activities. These events promote an enhancement of the lipoperoxidation, as well as of *γ*-GT, myeloperoxidase, and caspase 3 activities. With respect to ERE, there were ATF6, IRE1, and GADD153 overexpressions with a reduction in mitochondrial activity and GSH^2^/GSSG ratio. We conclude that the endoplasmic reticulum stress might play a pivotal role in the activation of hypothyroidism-induced hippocampal cell death.

## 1. Introduction

Hypothyroidism is a clinical condition in which thyroid hormone (TH) release of the thyroid gland is low, and it is not enough to satisfy the demand of the tissues [1]. TH deficiencies lead to a broad spectrum of clinical manifestations, including neurologic symptoms like memory impairment, difficulty concentrating, and depression [2, 3]. These alterations are directly linked with the hippocampus, which is especially sensitive to thyroid hormone deprivation [4–6]. Several studies have shown that hypothyroidism leads to neuronal death in the hippocampus [7]. The entire mechanism is not still well understood, but it involves the presence of oxidative stress [6] and the activation of the N-methyl-D-aspartate receptor- (NMDAR-) mediated glutamate excitotoxicity [5]. However, the biological events mentioned above are probably an activation consequence of several mechanisms; one of them can be the endoplasmic reticulum stress (ERE). The ERE is defined as the accumulation of misfolded or not folded proteins and their aggregates in the lumen of this organelle. The ERE triggers an adaptive response called the unfolded protein response (UPR) which is developed to restore the ER homeostasis, and also it allows the cell to attenuate general translation. The endoplasmic reticulum increases the synthesis of chaperones and other proteins involved in the UPR and eliminates the defective proteins by the endoplasmic reticulum-associated protein degradation (ERAD) mechanism [8]. When ERE is not alleviated, it becomes chronic and leads to the activation of caspase 12-activated cell death [9]. On the other hand, it has been demonstrated that nutrient deprivation (mainly oxygen and glucose) is one of the most potent ERE triggers [10], since it impairs N-linked protein glycosylation and diminishes the ATP obtaining. The processes mentioned before lead to a decrease in the amount of energy available to maintain the oxidizing environment needed to the correct formation of disulfide bonds and the proper folding of proteins in the endoplasmic reticulum (ER). Thus, the ERE triggers lead to ER overload of misfolded and not folded proteins and the consequent activation of the UPR[11].

A relation between hypothyroidism and ERE has not yet been studied. However, it is well known that hypothyroidism causes a reduction in the brain blood flow [12], and also it decreases the expression of glucose transporter GLUT1 on the blood-brain barrier [13], which can lead to a decrease in the brain glucose input. Also, hypothyroid subjects usually present with insulin resistance [14, 15], a condition that might provoke a reduction in glucose intake from neurons and glia, especially in the hippocampus, where there is a high expression of insulin-dependent GLUT4 transporter [16]. Therefore, we believe that there must be an association between the activation of endoplasmic reticulum stress and hypothyroidism-caused neuronal damage in the hippocampus. This study is aimed at evaluating the temporal participation of the endoplasmic reticulum stress (ERE) in hippocampal neurons of adult hypothyroid rats and its association with the oxidative stress events.

## 2. Methods

### 2.1. Animals and Housing and Experimental Design

Forty-eight male Wistar rats (200–250 g) of our care facilities were kept in acrylic cages (80 × 30 × 20 cm) in a cooled-regulated room (20 ± 1°C) with water and food ad libitum, light cycles of 12/12 h, and relative humidity of 40–60%. All procedures were realized according to Mexican laws and codes in the seventh title of the General Law of Health regarding health research and NOM-062-ZOO-1999 for the handle of laboratory animals, and these procedures were also approved by the ENCB-IPN Bioethics committee (CEI-ENCB-021/2014).

Animals were initially divided into two groups: hypothyroid (Hypo; *n* = 24) and euthyroid (Eu; *n* = 24). Hypothyroidism was induced by a surgical removal of the thyroid gland with reimplantation of the parathyroid gland in the pectoral muscle, as previously described by Pineda-Reynoso et al. [17]. A false thyroidectomy, with the same surgical and postsurgical cares, was performed on euthyroid animals. After surgery, each group was subdivided into the third week and fourth week subgroups, according to the times they were sacrificed.

### 2.2. Thyroid State Determinations

Rectal temperature of all groups was measured three times a week at the same hour in the morning (0900 h), using a digital thermometer (BD Diagnostics), just like body weight. Both variables were used as indirect measures of the thyroid state of rats, while thyroid hormones (T_3_ and T_4_) were quantified in the serum at the different times of sacrifice by ELISA, using commercial kits (Diagmex, México), and were used to establish thyroid state of rats at that time.

### 2.3. Sacrifice of Animals and Sampling

After the different test times, animals were beheaded. Immediately, the blood and the brains were obtained and separated. Four brains of each group were fixed in 4% PBS paraformaldehyde (PFH), while the hippocampus was dissected from the remaining eight brains. The hippocampus was immediately frozen and stored at −70°C until use.

### 2.4. General Preparation of the Samples

The dissected hippocampus was homogenized in 500 *μ*L·10 mM phosphate buffer (pH 7.4) and was used to perform all the biochemical determinations, the Western blot, and the mitochondrial function and caspase 3 activity assays.

### 2.5. Biochemical Determinations

The quantification of oxidative stress marker reactive oxygen species (ROS) and the lipid peroxidation assay were performed as previously described [18, 19], nitrites were assessed by Griess reaction, as previously described by Sastry et al. [20], and myeloperoxidase (MPO) activity was assessed by Fietz et al.'s method [21].

The activity of antioxidant enzymes was assessed by spectrophotometric techniques. For catalase activity, we followed the spectrophotometric method previously described by Cano-Europa et al. [22], the glutathione peroxidase (GPX) activity was measured by Hafeman et al.'s technique [23], total superoxide dismutase (SOD) activity was assessed by Crapo et al.'s method [24], and glutathione reductase (GR) activity was measured using the method proposed by Askelöf et al. [25].

The gamma-glutamyl transpeptidase (*γ*-GT) activity was determined by a commercial kit (*γ*-GT-LQ, Spinreact, Spain), according to the manufacturer's instructions.

The markers of redox environment reduced glutathione (GSH) and oxidized glutathione (GSSG) and were assessed by a spectrofluorometrical method, as previously described [22]. The GSH^2^/GSSG ratio was assessed according to Schafer and Buettner [26].

### 2.6. Western Blot Assay

Western blot samples were prepared as follows: one hundred microliters of homogenate was taken and mixed in Eppendorf tubes containing 30 *μ*L of a Complete Protease Inhibitor Cocktail® (Sigma-Aldrich, St. Louis Missouri, USA) in lysis buffer, and 130 *μ*L of charge buffer was added. The samples were homogenized in a vortex and then placed in a boiling water bath for 3 minutes. The samples were kept at −20°C until use.

Endoplasmic reticulum stress markers, IRE1 (Abcam, UK; ab37073), ATF6*α*, GADD34, GADD153, XBP-1 (Santa Cruz Biotechnology, Dallas, Texas, USA; sc-22799, sc-8327, sc-575, and sc-7160, resp.), ATF4 (Biorbyt, Cambridge, UK; orb-129518), and caspase 12 (Millipore, Billerica, Massachusetts, USA AB3613), were determined by Western blot analysis. Briefly, 50 *μ*g of protein was charged in 10% polyacrylamide gels and separated by electrophoresis. After that, proteins of gels were electrotransferred to PVDF membranes in a semidry chamber. Transferred membranes were blocked for 1 h under constant stirring, in blocking buffer (PBST; 0.05% tween 20 in saline phosphate buffer containing 5% low-fat milk Svelty®). Blocked membranes were incubated overnight in blocking buffer containing the primary antibodies diluted at 1 : 500 (ATF6*α*, GADD34, GADD153, XBP-1, and caspase 12), 1 : 1000 (IRE1), and 1 : 2000 (CREB2) at 4°C. After incubation, membranes received three 20 min washes with fresh PBST. Washed membranes were then incubated in blocking buffer containing 1 : 1000 diluted secondary antibody (HPR-conjugated goat anti-rabbit or rabbit anti-goat; Life technologies, Rockford, Illinois, USA; 65-6120 and 611620, resp.) for 1 h at room temperature, under constant stirring. Membranes were washed again, and finally, protein bands were revealed in photographic plates (JUAMA, México) using Luminata™ Forte® (Millipore, Billerica, Massachusetts, USA). Protein *β*-actin expression was used as a charge control and constitutive protein (Santa Cruz Biotechnology, Dallas Texas, USA; sc-1615, dilution: 1 : 4000). Optical density (OD) of all the bands was analyzed by ImageJ program version 1.51p (NIH, Bethesda, Maryland, USA), according to program specifications. Protein OD is expressed as a protein/*β*-actin index.

### 2.7. Immunofluorescence Assay

PFH-fixed brains were processed by a conventional paraffin-embedded technique. Paraffin cubes were cut in a microtome to obtain 5 *μ*m tissue laminae. The hippocampus region was localized at −1.8 and −2.3 with respect to Bregma, according to Paxinos and Watson [27]. We used the immunofluorescence test to detect the expression of ERE protein peIF2*α*. Briefly, tissue sections were deparaffinized and blocked with 1% bovine albumin (Sigma-Aldrich) in Tris-saline buffer (TBST) (pH 7.6), for one hour. After that, the slices were incubated with 1 : 200 diluted anti-peIF2*α* antibody (Santa Cruz Biotechnology, Dallas, Texas, USA; sc-12412) at 4°C overnight. After incubation, we performed three 10 min TBST washes. Then, the slices were incubated with a donkey anti-goat-FITC-coupled antibody (1 : 5000, Abcam, UK; ab8861) for one hour in darkness. Tissue laminae were washed again, and the immunofluorescence was detected with a fluorescence microscopy. Images obtained were analyzed with the program ImageJ version 1.51p.

### 2.8. Mitochondrial Function Test and Caspase 3 Activity

Mitochondrial activity was measured as previously described by Elinos-Calderón et al. [28] modified by us, as we used p-iodonitrotetrazolium violet (INT) instead of tetrazolium blue. Mitochondrial function is expressed as % of INT reduction, with respect to the euthyroid group.

Caspase 3 activity was assessed by using a commercial colorimetric assay kit (Millipore, Billerica, Massachusetts, USA; APT165), according to the manufacturer's instructions. Caspase activity is expressed as *μ*mol of PNA released/mg protein/min.

### 2.9. Statistical Analysis

All data were analyzed through the Sigma Stat program version 3.5 (Systat Software Inc., San Jose, California, USA) and were presented as mean ± standard error of the mean. A repeated measures two-way analysis of variance (RM two-way ANOVA) was performed for rectal temperature, while the rest of the results were analyzed by two-way ANOVA. All analyses considered the thyroid state (euthyroid or hypothyroid) and time (third week or fourth week) as factors, followed by a Student-Newman-Keuls post hoc test. Values that presented a *P* < 0.05 were considered statistically different.

## 3. Results

### 3.1. Effect of Thyroidectomy on Rectal Temperature and Serum T_3_ and T_4_ Levels

**Figure 1:**
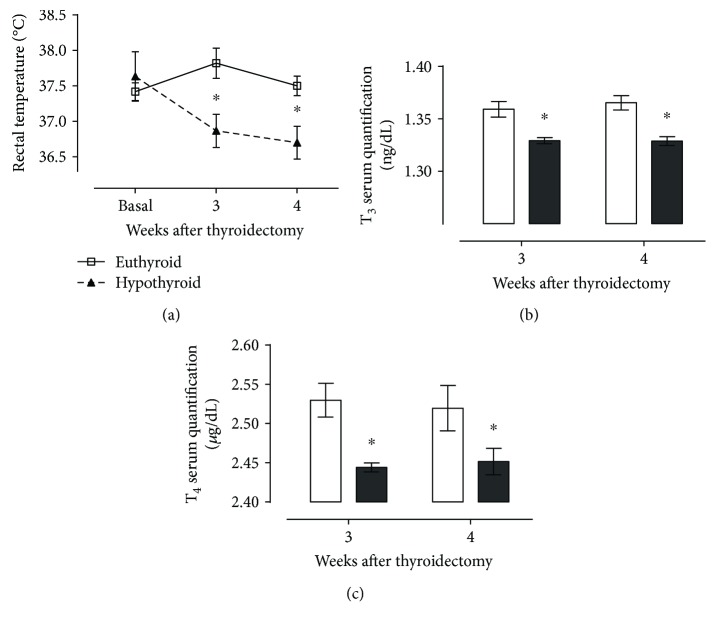
Effect of thyroidectomy on rectal temperature (a), T_3_ serum quantification (b), and T_4_ serum quantification (c) of rats at different times after surgery. Data are expressed as the mean ± SEM. ^∗^*P* < 0.05 versus the euthyroid group (*n* = 24).

Figure 1 shows that thyroidectomy decreases rectal temperature (Figure 1(a)) and serum thyroid hormones T_3_ (Figure 1(b)) and T_4_ (Figure 1(c)) at both third and fourth weeks, with respect to sham animals. These results allowed us to be sure that thyroidectomized rats presented signs of hypothyroidism.

### 3.2. Hypothyroidism Causes Oxidative Stress and Modifies the Antioxidant Enzyme Activity and the Redox Environment

**Figure 2:**
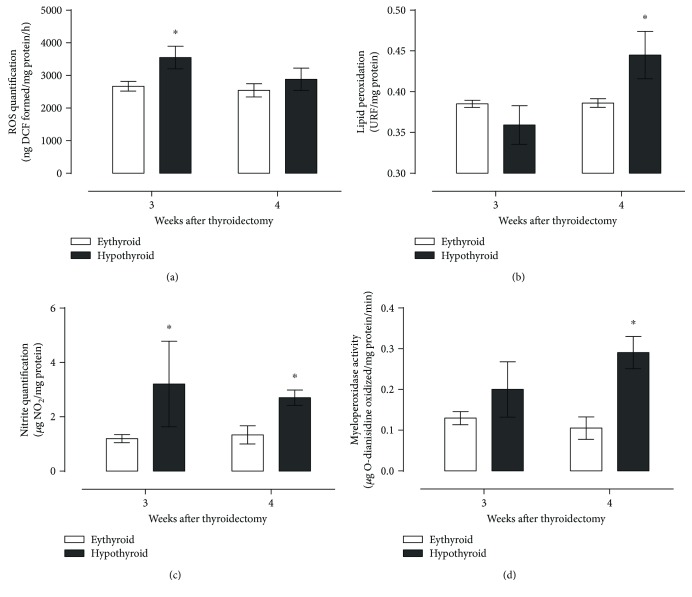
Effect of hypothyroidism on oxidative stress markers reactive oxygen species (a), lipid peroxidation (b), nitrite quantification (c), and myeloperoxidase activity (d). Data are expressed as the mean ± SEM. ^∗^*P* < 0.05 versus the euthyroid group (*n* = 6).

**Figure 3:**
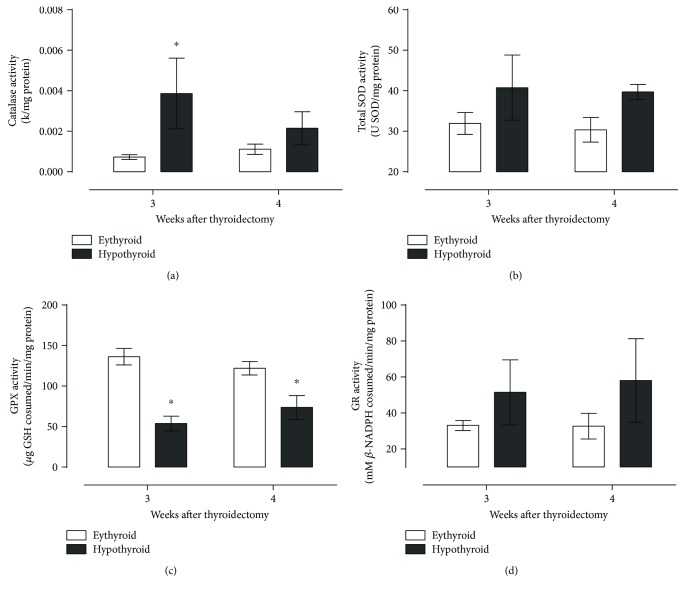
Activity of antioxidant enzymes catalase (a), superoxide dismutase (b), glutathione peroxidase (c), and glutathione reductase (d) in the hippocampus of thyroidectomized rats. Data are expressed as the mean ± SEM. ^∗^*P* < 0.05 versus the euthyroid group (*n* = 6).

Figure 2 shows that hypothyroid rats presented an increased production of ROS (Figure 2(a)) and nitrites (Figure 2(c)) at the third week, followed by a rise in lipid peroxidation (Figure 2(b)) and a higher myeloperoxidase activity at the fourth week postthyroidectomy (Figure 2(d)). Increased oxidant products were not successfully neutralized by antioxidant enzymes catalase, SOD, GPX, and GR (Figures 3(a)–3(d), resp.), since only catalase activity increased at the third week and GPX activity was diminished at both times evaluated.

Table 1 data shows a reduction in GSH concentration with a GSH^2^/GSSG ratio reduction for hypothyroid animals at the fourth week. These modifications could not be compensated by the progressive increased *γ*-GT activity.

All modifications in the quantification of oxidative products, the antioxidant enzyme activities, and redox environment marker modifications demonstrate the presence of oxidative stress at the fourth week and the diminished capability of the cell to maintain the redox environment in hypothyroid animals.

### 3.3. Hypothyroidism Causes Endoplasmic Reticulum Stress

To probe that hypothyroidism may induce endoplasmic reticulum stress in the hippocampus, we determined the expression of key molecular players involved in PERK, ATF6, and IRE1 branches of the UPR.

**Figure 4:**
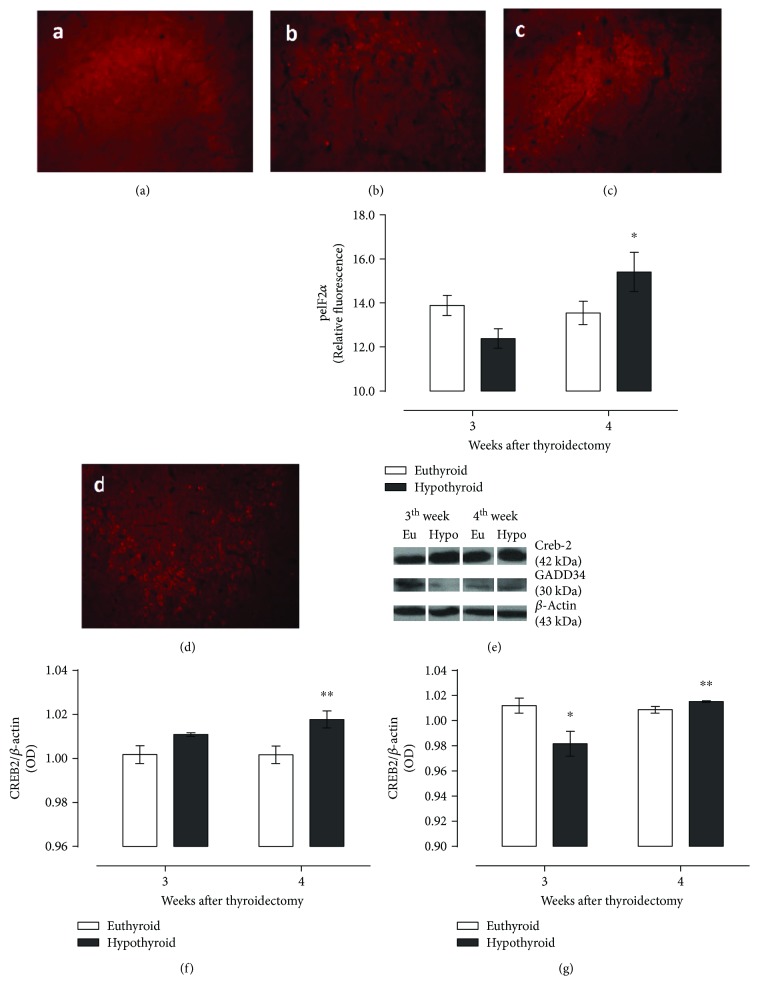
Photomicrography of the hippocampus of euthyroid rats (a, c) and thyroidectomized rats (b, d) at the third and fourth weeks after surgery, expression of PERK substrates pEIF2*α* (e) and CREB2 (f), and the PERK signal inhibitor GADD34 (g) in the hippocampus of euthyroid and thyroidectomized rats. Values are expressed as the mean ± SEM. ^∗^*P* < 0.05 versus the euthyroid group; ^∗∗^*P* < 0.05 versus the third week hypothyroid group (*n* = 3).

Figure 4 integrates the results of the expression of the PERK branch, which was measured through the expression of peIF2*α* (Figures 4(a)–4(e)), CREB2 (Figure 4(f)), and GADD34 (Figure 4(g)). Apparently, this response is at least not fully activated, due to phosphorylation of eIF2*α* which was probably inhibited by a protein different from GADD34, since the expression of both markers is not increased at any evaluated time, with respect to euthyroid animals, and just presented a light increase compared to the response in the hypothyroid group at the third week. GADD34 expression is also diminished at the third week if we compare such expression with that of euthyroid animals at the same evaluated time. Besides the nondifferent phosphorylation of eIF2*α*, CREB2 expression was higher than that of its euthyroid control at the fourth week, indicating the partial activity of PERK response.

**Figure 5:**
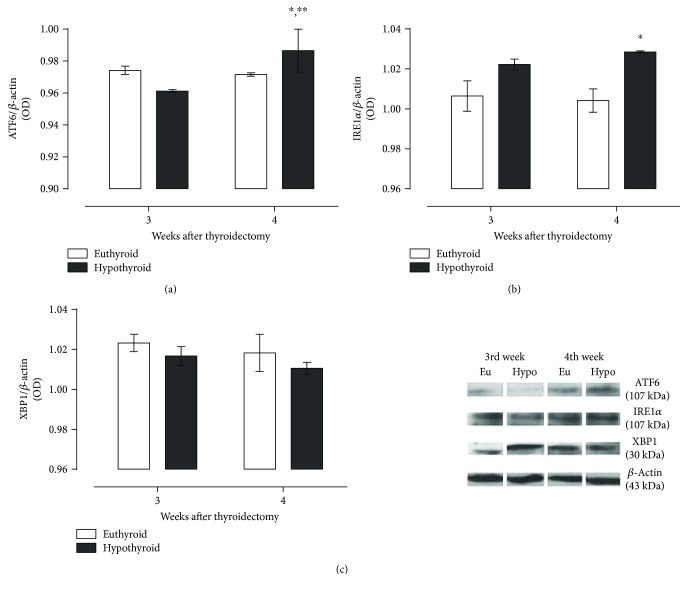
Expression of ATF-6 (a), IRE-1*α* (b), and its product of activation XBP-1 (c) in the hippocampus of thyroidectomized rats at different periods of time. Data are expressed as the mean ± SEM. ^∗^*P* < 0.05 versus the euthyroid group; ^∗∗^*P* < 0.05 versus the hypothyroid group at the third week (*n* = 3).

The expression of the second and third branches of the UPR, mediated by ERE sensors ATF6 and IRE1*α*, showed an increase in the hypothyroid group at the fourth week, as we show in Figures 5(a) and 5(b). The expression of XBP1 (Figure 5(c)) was not modified during hypothyroidism, indicating that ribonuclease activity of IRE1*α* was not increased as a response for this condition.

### 3.4. Endoplasmic Reticulum Stress Leads to Hippocampal Neuron Death

**Figure 6:**
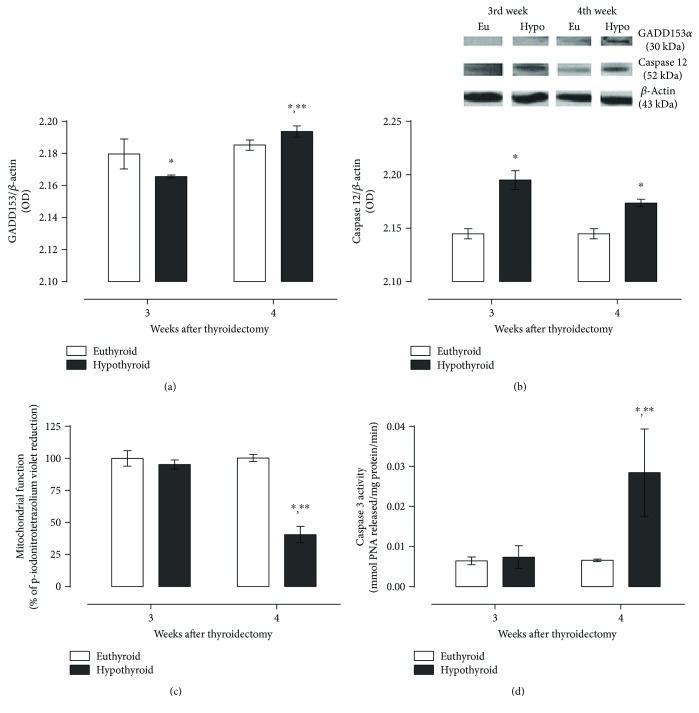
Expression of proapoptotic proteins GADD153 (a) and caspase 12 (b), mitochondrial function (c), and caspase 3 activity (d) in the hippocampus of thyroidectomized rats at different periods of time. Data are expressed as the mean ± SEM. ^∗^*P* < 0.05 versus the euthyroid group; ^∗∗^*P* < 0.05 versus the hypothyroid group at the third week (for (a) and (b) (*n* = 3) and for (c) and (d) (*n* = 6)).

Due to the higher expression of CREB2 and ATF6 in the hypothyroid group at the fourth week, we evaluated the expression of GADD153 (Figure 6(a)) and found that this protein was less expressed in hypothyroid animals at the third week but had a rise at the fourth week.

Given the proapoptotic function of GADD153, we also evaluated some ER-related death proteins and the mitochondrial activity of hippocampal cells and found that the hypothyroid state induces the expression of the ER membrane-associated caspase 12 at both times evaluated (Figure 6(b)). Mitochondrial function was not different between euthyroid and hypothyroid rats at the third week but presented a 50% decay in hypothyroid animals at the fourth week (Figure 6(c), indicating the damage in this organelle. Finally, we evaluated the activity of the effector protein caspase 3 that, as we expected, presented a practically three-fold increase in hypothyroid animals at the fourth week (Figure 6(d)), demonstrating that hippocampal neurons activated cell death mechanisms mediated, at least partially, by the activation of ER caspase 12.

## 4. Discussion

Several studies attribute hypothyroidism-induced neuronal death in the hippocampus to glutamate excitotoxicity mediated by N-methyl-D-aspartate receptors (NMDAR). This hypothesis is supported because hypothyroidism causes a reduction in Na^+^/K^+^ ATPase expression and activity in the membrane of hippocampal neurons [29]. This event probably modifies the neuronal rest potential which finally leads to the activation of NMDA receptors that import calcium ions to the neuron cytoplasm and activate calcium-sensitive kinases, inducing mitochondrial damage and leading to apoptosis [30]. Besides that this is one of the most accepted mechanisms of hypothyroidism-induced hippocampal neuronal death, it cannot be the only one, or at least, not the primary inducer for several reasons, one of which is that other works have found that glutamate synthesis and release are diminished in hypothyroidism [31, 32]. Based on the evidence described before, we developed the hypothesis that there had to be another mechanism involved that occured first but also promoted the development of oxidative stress and the excitotoxicity events found in previous works [5, 6]. This missed mechanism can be the endoplasmic reticulum stress, which has been established as a common trigger to cell death in several pathologies (and also in normal aging), that includes the development of oxidative stress [33]. The reasons why we thought ERE might be involved are based on the contributions of many studies, where hypothyroidism has been related to peripheral glucose metabolism impairment [14, 34–37] and the presence of insulin resistance [14, 15, 36]. In the case of the brain, glucose concentration in cerebral spine fluid or any brain regions had not been assessed; however, glucose transportation from the blood-brain barrier is decreased during hypothyroidism, as previously reported by Mooradian et al. who found a significative reduction in both mRNA and 55 kDa GLUT1 isoform [13]. Also, other reports have shown that global and regional cerebral blood flux to the hippocampus and other memory-, motor-, attention-, and cognitive-related structures is diminished in hypothyroid patients [12]. As it is well known, brain glucose metabolism depends on thyroid function, which is why hypothyroid patients present with brain hypometabolism, a phenomenon that has been linked with the impairment of memory and cognition [38, 39]. Also, insulin resistance can play an essential role in hypothyroidism-induced cognitive damage, since the normal hippocampus expresses high amounts of GLUT4 transporters, and insulin signaling has recently demonstrated to be necessary for hippocampal-mediated memory processes because it increases hippocampal metabolism [40, 41]. Given the impaired glucose delivery to the brain, the diminished glucose intake and metabolism in hypothyroid hippocampal neurons, and the relationship between glucose deprivation and ERE, we determined the expression of markers from the three branches of the UPR and the presence of oxidative stress in the hippocampus of hypothyroid rats, three and four weeks after thyroidectomy. We found that PERK branch of the UPR might be partially activated at the third week after thyroidectomy because CREB2 expression was higher in hypothyroid homogenates. The abovementioned is relevant because PERK is the first sensor that activates in the UPR and its downstream activates transcription factor CREB2, which primary interacts with c-AMP response elements in DNA to initiate the transcription of genes that codify chaperones, sensors, and other proteins involved in the UPR [42]. Also, CREB2 can interact with the GADD153 promoter (due to its relative homology with c-AMP response elements) and activate its translation [43]. One finding that surprised us was that hypothyroid has no peIF2*α* overexpression. This result might be explained by the activation of sirtuin1 (SIRT1), a molecule that can deacetylate eIF2*α* and avoid its phosphorylation. Thus, the amount of peIF2*α* is reduced [44]. Also, SIRT1 binds to GADD34 and increases its function, promoting the dephosphorylation of peIF2*α* and SIRT1. Dephosphorylated SIRT1 promotes a positive feedback loop on GADD34, and it enhances dephosphorylation of peIF2*α*, attenuating general transcription (which could explain the lack of difference in the expression of GADD34 between the hypothyroid and euthyroid groups) [45]. In this model of hypothyroidism, attenuation of the general transcription is not successful in maintaining the prosurvival environment into the cell, allowing the activation of proapoptotic pathways partially mediated by both PERK and ATF6 branches, since CREB2 and ATF6 induce the transcription of GADD153 [46] and this protein can activate cell death through several ways, including the repression of the *Bcl2* gene and also the direct inhibition of Bcl-2, an antiapoptotic protein that binds proapoptotic proteins Bax and Bim and inhibits them [47]. Also, GADD153 can increase the endoplasmic reticulum oxireductin 1 (ERO1) activity and expression [48]. ERO1 is a protein that transfers electrons to oxygen and leads to the formation of H_2_O_2_ in the normal process of sulfide-bound formation on the ER, but, that in a maladaptive UPR, can act as an important source of H_2_O_2_ [49]. On the other hand, ERO1 has been linked to calcium cytotoxicity, since it can activate the inositol (1,4,5)-trisphosphate receptor type 1 (IP3R1), leading to the massive release of ER calcium to the cell cytoplasm that can activate calcium/calmodulin-dependent kinase II (CAMKII) which might induce the NADPH oxidase 2 (NOX2), a complex of enzymes in which the primary function is to produce ROS, through the exchange of electrons across membranes to reduce oxygen and form superoxide and other ROS [50]. Given the high expression of these oxidases in the immune cells, especially in phagocytes, and the increased astrogliosis observed in hypothyroidism [51], the activation of NOX2, along with other enzymes like MPO, might be one of the primary sources of ROS and contribute importantly to the establishment of oxidative stress.

For its part, IRE1 performs its prosurvival function through the formation of XBP1s, which heterodimerizes with nuclear factor Y (NF-Y) and increases translation of proteins involved in the UPR and the endoplasmic reticulum-associated protein degradation (ERAD) [52]. Our results indicate that IRE1 is probably activating its proapoptotic branch. According to that, the kinase dominium of this sensor binds to TRAF2 and recruits to ASK1, leading to the activation of JNK, that phosphorylates ER Bcl-2 and inhibits it, allowing Bax, Bak, and Bim to exert their proapoptotic functions at both ER and mitochondria [53]. It is important to underline that Bax and Bak can activate IRE1 when they directly interact with this sensor [54] and may be an enhancer of this proapoptotic pathway. Also, IRE1*α*-TRAF2 complex, joined to the calcium released from ER and the direct action of Bax/Bak, can lead to the activation of ER membrane caspase 12. This caspase can activate mitochondrial-released caspase 9, and both activate effector caspase 3 [9], which we firmly believe happens in hypothyroidism-induced neuronal death in the hippocampus.

Finally, we conclude that endoplasmic reticulum stress is probably the primary mechanism that leads to the death of hippocampal neurons seen in hypothyroidism. We cannot exclude glutamate excitotoxicity as an important hypothyroidism-induced mechanism of neuronal death, and, in fact, we propose it as the next step in this intricate death signaling pathways. More studies will be needed to better elucidate the final death mechanism activated during hypothyroidism, since it can implicate the activation of other pathways than apoptosis and include the participation of the immune system, through the inflammatory response.

## Figures and Tables

**Table 1:** Redox environment alterations in the hippocampus of thyroidectomized rats at different periods of time.

	3rd week		4th week	
	Euthyroid	Hypothyroid	Euthyroid	Hypothyroid
GSH (*μ*g GSH/mg protein)	426.66 ± 36.16	452.02 ± 41.76	447.59 ± 32.35	332.94 ± 36.16^∗^^,^^∗∗^
GSSG (*μ*g GSSG/mg protein)	1.33 ± 0.25	1.51 ± 0.22	1.40 ± 0.19	1.42 ± 0.19
Index GSH^2^/GSSG	145318.53 ± 27632.49	130329.80 ± 27632.49	155529.19 ± 23930.43	76109.75 ± 27632.49^∗^^,^^∗∗^
*γ*-Glutamyl transpeptidase activity (UgGT/min/mg protein)	1.27 ± 0.37	3.04 ± 0.59^∗^	1.27 ± 0.37	5.34 ± 0.59^∗^^,^^∗∗^

Values are expressed as the mean ± SEM. ^∗^*P* < 0.05 versus the euthyroid group; ^∗∗^*P* < 0.05 versus the third week hypothyroid group (*n* = 6).
